# Comparison of the association intensity of creatinine and cystatin C with hyperphosphatemia and hyperparathyroidism in patients with chronic kidney disease

**DOI:** 10.1038/s41598-023-31048-2

**Published:** 2023-03-08

**Authors:** Byungju Min, Sung-Ro Yun, Se-Hee Yoon, Jong-Dai Kim, Wan Jin Hwang, Won Min Hwang, Yohan Park

**Affiliations:** 1 Division of Nephrology, Department of Internal Medicine, Konyang University Hospital, Gwanjeodong-Ro 158, Seo-Gu, Daejeon, 35365 Republic of Korea; 2 Division of Endocrinology, Department of Internal Medicine, Konyang University Hospital, Gwanjeodong-Ro 158, Seo-Gu, Daejeon, 35365 Republic of Korea; 3 Konyang University Myunggok Medical Research Institute, Gwanjeodong-Ro 158, Seo-Gu, Daejeon, 35365 Republic of Korea

**Keywords:** Nephrology, Kidney

## Abstract

Herein, we compared the association intensity of estimated glomerular filtration rate (eGFR) equations using creatinine (Cr) or cystatin C (CysC) with hyperphosphatemia and secondary hyperparathyroidism occurrence, which reflect the physiological changes occurring during chronic kidney disease (CKD) progression. This study included 639 patients treated between January 2019 and February 2022. The patients were divided into low- and high-difference groups based on the median value of the difference between the Cr-based eGFR (eGFR_Cr_) and CysC-based eGFR (eGFR_CysC_). Sociodemographic and laboratory factors underlying a high difference between eGFR_Cr_ and eGFR_CysC_ were analyzed. The association intensity of eGFR_Cr_, eGFR_CysC_ and both Cr- and CysC-based eGFR (eGFR_Cr-CysC_) was compared using the area under the receiver operating characteristic curve (AuROC) values for hyperphosphatemia and hyperparathyroidism occurrence in the overall cohort and the low- and high-difference groups. Age > 70 years and CKD grade 3 based on eGFR_Cr_ were significant factors affecting the high differences. eGFR_CysC_ and eGFR_Cr-CysC_ showed higher AuROC values than that of eGFR_Cr_, especially in the high-difference group and in patients with CKD grade 3. Our results show that CysC should be evaluated in patients with significant factors, including age > 70 years and CKD grade 3, to accurately assess kidney function to better determine the physiological changes in CKD progression and predict prognosis accurately.

## Introduction

Creatinine (Cr) is the most widely used indicator to estimate kidney function, and assessing the estimated glomerular filtration rate (eGFR) using the serum Cr is recommended for assessing kidney function in the clinical field^1,2^. However, since serum Cr can be affected by various factors, including age, muscle mass, race, diet, drugs, and renal tubular secretion, its ability to reflect kidney function alone is limited^3^. Cystatin C (CysC) is constantly produced in all nucleated cells, and is excreted by the kidneys. The measurement of CysC is recommended as an alternative indicator of kidney function as it is less affected by other factors than serum Cr is^4–6^. However, CysC is affected by smoking, inflammation, adiposity, certain malignancies, and glucocorticoid use; therefore, it also has some limitations as an indicator of kidney function^7–9^.

The Chronic Kidney Disease-Epidemiology Collaboration (CKD-EPI) presented equations for Cr-based eGFR (eGFR_Cr_), CysC-based eGFR (eGFR_CysC_), and both Cr- and CysC-based eGFR (eGFR_Cr-CysC_)^10^. Many previous studies have reported that the eGFR_Cr-CysC_ has the highest accuracy as a direct measurement of GFR (mGFR) and that it is also the most accurate index in patients with diabetes, liver cirrhosis, or solid tumors^4,11–13^. Serum Cr tends to be high in Black people regardless of their kidney function; therefore, race is considered in previous CKD-EPI equations containing serum Cr. However, in 2021, the CKD-EPI proposed new equations regardless of race, and the new eGFR_Cr-CysC_ equation showed higher accuracy with mGFR compared to those of previous equations^10^.

Hyperphosphatemia and secondary hyperparathyroidism are common clinical features of chronic kidney disease (CKD)^14^. As CKD gradually progresses to an advanced stage, the prevalence of hyperphosphatemia and secondary hyperparathyroidism is increased via various mechanisms, including a decrease in renal phosphorus excretion and increase in bone resorption. In other words, hyperphosphatemia and secondary hyperparathyroidism are considered to be physiological changes following CKD progression, and are associated with cardiovascular complications and mortality in CKD^15–17^.

Many studies have reported on the accuracy of the three eGFR equations (eGFR_Cr_, eGFR_CysC_, and eGFR_Cr-CysC_) and mGFR. However, the association of each of the eGFR equations for the occurrence of hyperphosphatemia and secondary hyperparathyroidism has not yet been elucidated. Therefore, the present study compared the association intensity of eGFR_Cr_, eGFR_CysC,_ and eGFR_Cr-CysC_ with the occurrence of hyperphosphatemia and hyperparathyroidism with hyperphosphatemia in patients with CKD, especially in patients with a high difference between the eGFR_Cr_ and eGFR_CysC_.

## Results

### Comparison of baseline characteristics between the low- and high-difference groups

Patients were divided into low- and high-difference groups based on the median value of the difference between the eGFR_Cr_ and eGFR_CysC_ (|eGFR_Cr_-eGFR_CysC_|: < 6.353 ml/min/1.73 m^2^ and ≥ 6.353 ml/min/1.73 m^2^, respectively). Table 1 shows the baseline characteristics of the low- and high-difference groups. The proportion of patients aged > 70 years with CKD grade 3 based on eGFR_Cr_ was significantly higher in the high-difference group than in the low-difference group. The proportion of males and the prevalence of hypertension (HTN) were lower in the high-difference group compared with those in the low-difference group, with marginal statistical significance (*P* = 0.059 and *P* = 0.061, respectively). The high-difference group had significantly higher eGFR_Cr_ and lower eGFR_CysC_ values than those of the low-difference group. The eGFR_Cr-CysC_ showed no significant difference between the two groups. Most laboratory test findings, including the urine protein/creatinine ratio (PCR), showed no significant difference between the two groups, except for serum Cr and intact parathyroid hormone (PTH) levels.

**Table 1:** Comparison of the baseline characteristics between the low- and high-difference groups.

	Low-difference (n = 320)	High-difference (n = 319)	*P* value
Age > 70 years	172 (53.8%)	216 (67.7%)	< 0.001
Male (n, %)	204 (63.7%)	180 (56.4%)	0.059
CKD grade based on eGFR_Cr_			< 0.001
Grade 3 (n, %)	156 (48.8%)	235 (73.7%)	
Grade 4 (n, %)	164 (51.2%)	84 (26.3%)	
eGFR (ml/min/1.73 m^2^)			
eGFR_Cr_	31.90 ± 11.82	37.22 ± 9.74	< 0.001
eGFR_CysC_	29.73 ± 12.20	27.72 ± 10.37	0.025
eGFR_Cr-CysC_	31.14 ± 12.67	32.07 ± 10.41	0.311
Comorbidities			
HTN (n, %)	194 (60.6%)	170 (53.3%)	0.061
DM (n, %)	126 (39.4%)	109 (34.2%)	0.172
Laboratory findings			
Cr (mg/dl)	2.23 ± 0.74	1.81 ± 0.48	< 0.001
Cystatin C (mg/L)	2.19 ± 0.66	2.24 ± 0.65	0.348
Hemoglobin (g/dl)	11.25 ± 1.62	11.25 ± 1.83	0.992
Albumin (g/dl)	3.97 ± 0.40	3.93 ± 0.40	0.294
Uric acid (mg/dl)	6.09 ± 2.17	6.29 ± 2.27	0.261
CRP (mg/dl)	0.27 ± 0.76	0.32 ± 0.74	0.580
Calcium (mg/dl)	8.88 ± 0.59	8.87 ± 0.64	0.822
Inorganic phosphorus (mg/dl)	3.70 ± 0.71	3.72 ± 0.66	0.632
Intact PTH (pg/dl)	102.26 ± 85.19	83.81 ± 59.97	0.002
Urine PCR (g/g Cr)	1.56 ± 2.05	1.38 ± 1.93	0.271

Continuous variables are expressed as the mean ± standard deviation. Categorical variables are expressed as numbers (percentages).

*Ca* Calcium, *CKD* Chronic kidney disease, *Cr* Creatinine, *CRP* C-reactive protein, *DM* Diabetes mellitus, *eGFR* Estimated glomerular filtration rate, *eGFR*_*Cr*_ eGFR based on creatinine, *eGFR*_*CysC*_ eGFR based on cystatin C, *eGFR*_*Cr-CysC*_ eGFR based on both creatinine and cystatin C, *HTN* Hypertension, *PCR* Protein/creatinine ratio, *PTH* Parathyroid hormone.

### ***Distribution of the differences between eGFR***_***Cr***_*** and eGFR***_***CysC***_*** and scatter plot of eGFR***_***Cr***_*** and eGFR***_***CysC***_

**Figure 1:**
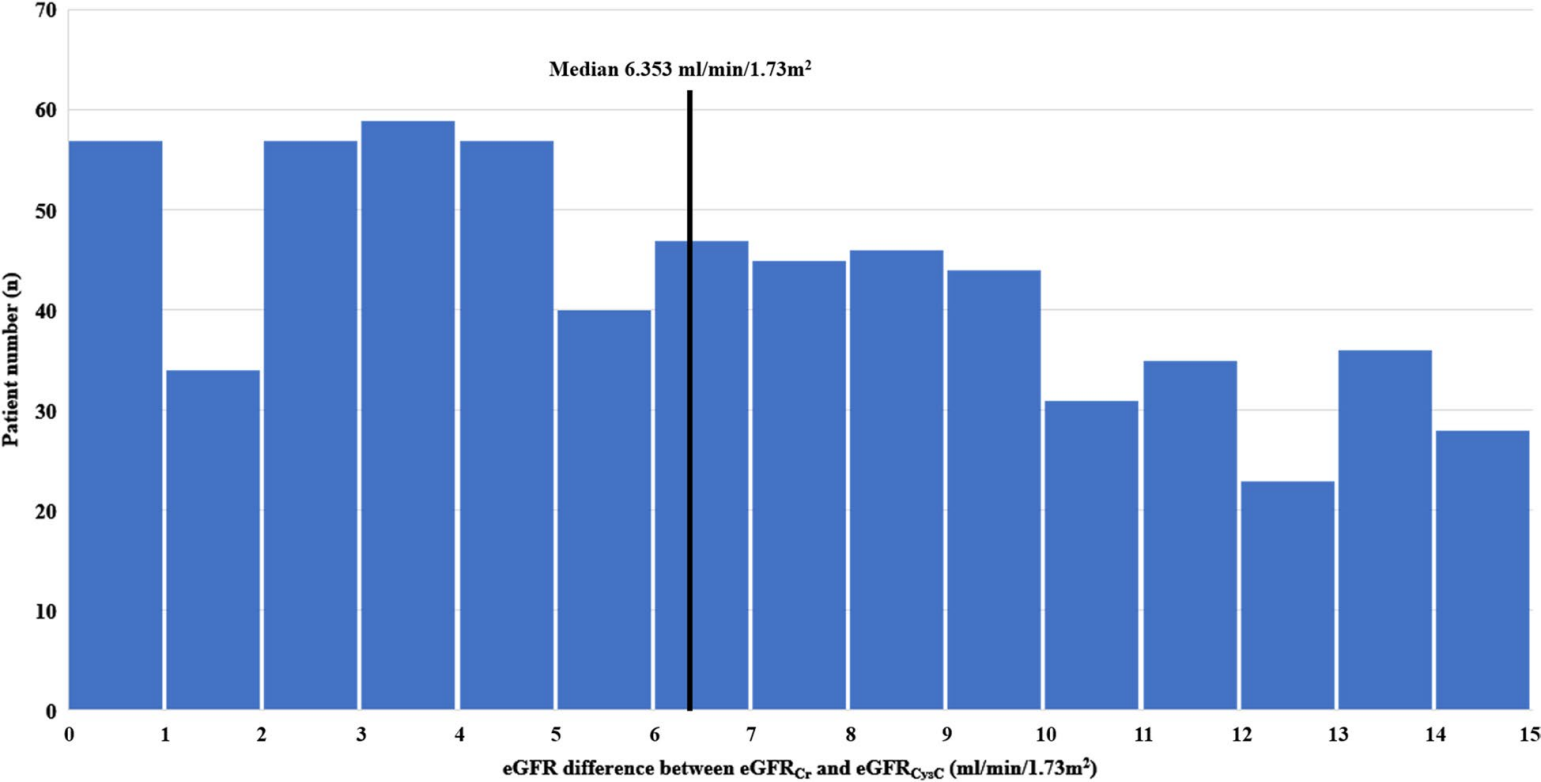
Distributions of differences between the eGFR_Cr_ and eGFR_CysC_. eGFR, estimated glomerular filtration rate; eGFR_Cr_, eGFR based on creatinine; eGFR_CysC_, eGFR based on cystatin C.

**Figure 2:**
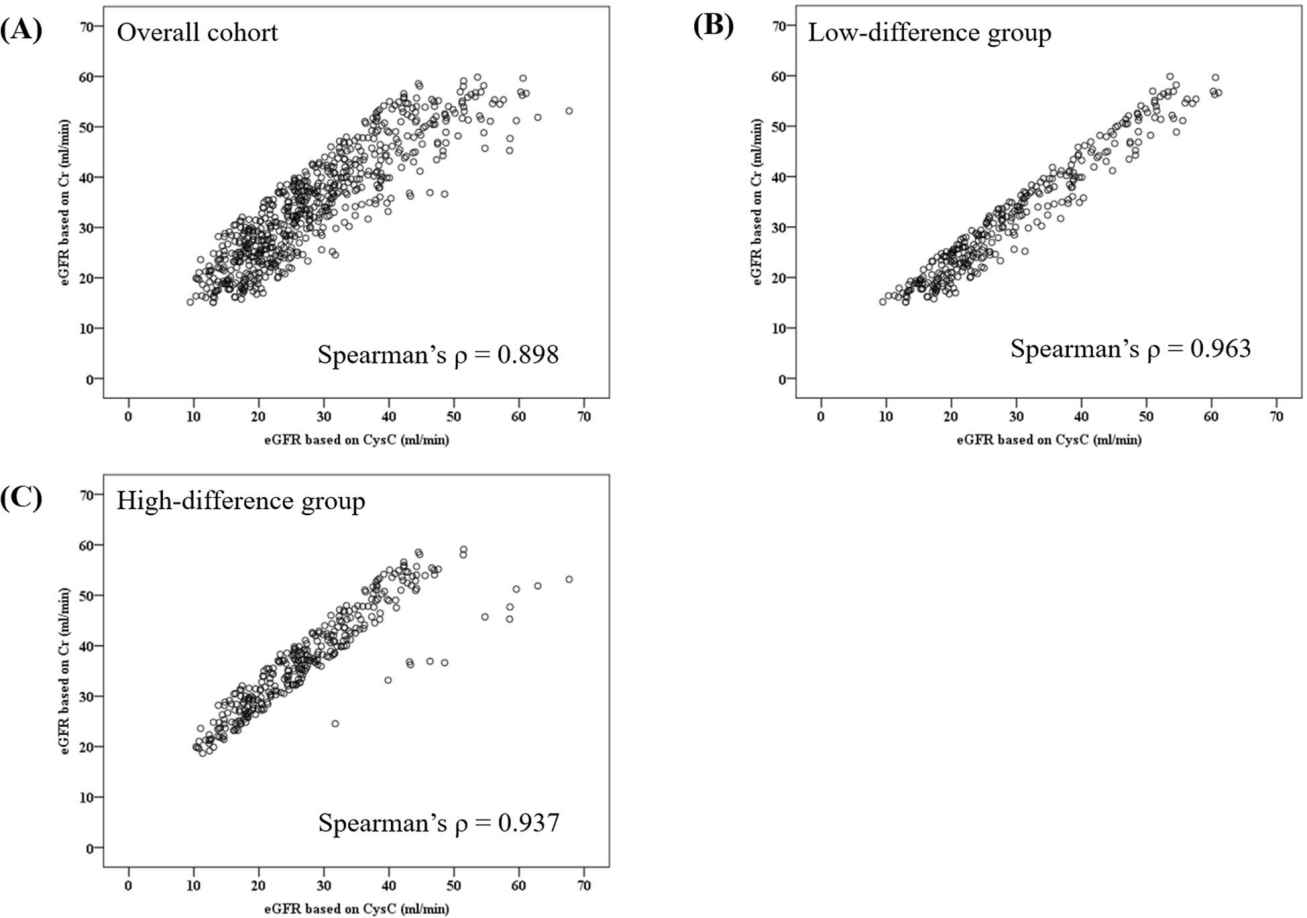
Scatter plot and correlation analysis of eGFR_Cr_ and eGFR_CysC_. Panels (**A**), (**B**), and (**C**) show the scatter plots of the overall cohort, low-difference group, and high-difference group. The Spearman’s correlation coefficient is noted on each scatter plot. eGFR, estimated glomerular filtration rate; eGFR_Cr_, eGFR based on creatinine; eGFR_CysC_, eGFR based on cystatin C.

Figure 1 shows the distribution of the differences between eGFR_Cr_ and eGFR_CysC_. The median value of the difference between eGFR_Cr_ and eGFR_CysC_ was 6.353 ml/min/1.73 m^2^, and the highest number of patients (n = 58) had an eGFR_Cr_ and eGFR_CysC_ difference of 3–4 ml/min/1.73 m^2^. Figure 2 shows the scatter plot of eGFR_Cr_ and eGFR_CysC_ in the overall cohort and in the low- and high-difference groups. All three groups showed a positive correlation between eGFR_Cr_ and eGFR_CysC_. The low-difference group had the highest correlation coefficient. In the scatter plot of the high-difference group; eGFR_Cr_ was greater than eGFR_CysC_ in most patients (307/319, 96.2%).

### ***Factors responsible for the high difference between eGFR***_***Cr***_*** and eGFR***_***CysC***_

Table 2 shows the results of a logistic regression analysis conducted to identify factors in baseline characteristics that affect the occurrence of high differences in |eGFR_Cr_-eGFR_CysC_|. Factors that showed statistically significant differences at baseline between the two groups were analyzed. CKD grade 3 based on the eGFR_Cr_ and age > 70 years were significant factors in the univariate analysis, with odds ratios (OR) of 3.179 (*P* < 0.001) and 2.011 (*P* < 0.001), respectively. CKD grade 3 and age > 70 years were also observed as independent factors for a high difference in |eGFR_Cr_-eGFR_CysC_|, with multivariate ORs of 3.191 (*P* < 0001) and 2.048 (*P* < 0.001), respectively.

**Table 2:** Logistic regression analysis of the high difference between the eGFR_Cr_ and eGFR_CysC_.

	Univariate OR (95% confidence interval)	Multivariate OR (95% confidence interval)
CKD grade 3 based on eGFR_Cr_ (Ref. CKD grade 4)	3.179 (2.113–4.783)	3.191 (2.210–4.606)
Male (Ref. Female)	1.195 (0.826–1.729)	–
Age > 70 years	2.011 (1.383–2.923)	2.048 (1.433–2.927)
HTN	0.846 (0.591–1.212)	–
Intact PTH	0.999 (0.996–1.002)	–
Urine PCR	1.054 (0.961–1.156)	–

Excluding patients with missing values, a total of 557 (87.2%) patients were included in multivariate logistic regression model.

*CKD* Chronic kidney disease, *eGFR* Estimated glomerular filtration rate, *eGFR*_*Cr*_ eGFR based on creatinine, *eGFR*_*CysC*_ eGFR based on cystatin C, *HTN* Hypertension, *OR* Odds ratio, *PCR* Protein/creatinine ratio, *PTH* Parathyroid hormone.

### Comparison of AuROC values of eGFR equations for hyperphosphatemia and hyperparathyroidism with hyperphosphatemia

**Figure 3:**
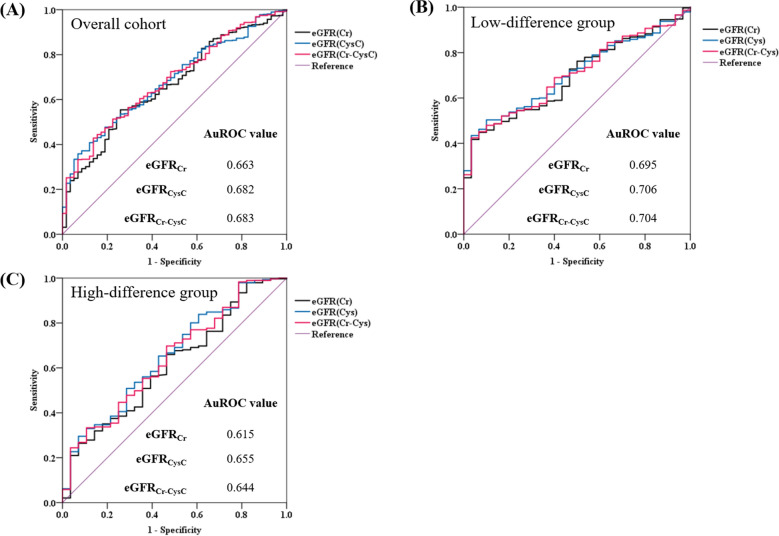
ROC curves and AuROC values of each eGFR equation for hyperphosphatemia. Panels (**A**), (**B**), and (**C**) show the ROC curves for each eGFR equation for the overall cohort, low-difference group, and high-difference group. The AuROC values of the eGFR equations are noted in each panel. AuROC, area under the ROC curve; eGFR, estimated glomerular filtration rate; eGFR_Cr_, eGFR based on creatinine; eGFR_CysC_, eGFR based on cystatin C; eGFR_Cr-CysC_, eGFR based on both creatinine and cystatin C; ROC, receiver operating characteristic.

Figure 3 shows the receiver operating characteristic (ROC) curves and area under the ROC curve (AuROC) values of the eGFR_Cr_, eGFR_CysC_, and eGFR_Cr-CysC_ for hyperphosphatemia occurrence in the overall cohort and in the low- and high-difference groups. In the overall cohort, the AuROC values of eGFR_CysC_ and eGFR_Cr-CysC_ were slightly higher than that of eGFR_Cr_ (0.682 for eGFR_CysC_ and 0.683 for eGFR_Cr-CysC_ vs. 0.663 for eGFR_Cr_). In the low-difference group, the AuROC values of eGFR_CysC_ and eGFR_Cr-CysC_ showed no significant difference from eGFR_Cr_. However, in the high-difference group, the AuROC values of eGFR_CysC_ and eGFR_Cr-CysC_ were higher than that of eGFR_Cr_, and the increase was more prominent in the high-difference group than in the overall cohort (0.655 for eGFR_CysC_ and 0.644 for eGFR_Cr-CysC_ vs. 0.615 for eGFR_Cr_).

**Figure 4:**
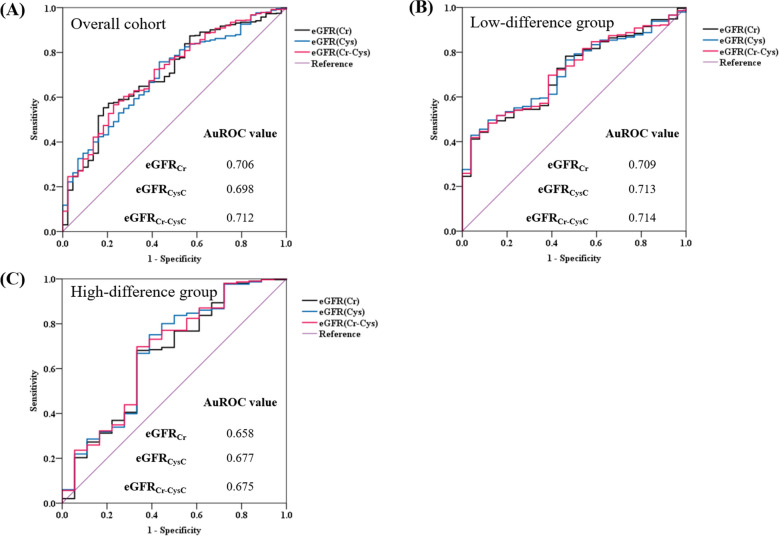
ROC curves and AuROC values of each eGFR equation for hyperparathyroidism with hyperphosphatemia. Panels (**A**), (**B**), and (**C**) show the ROC curves for each eGFR equation for the overall cohort, low-difference group, and high-difference group. The AuROC values of the eGFR equations are noted in each panel. AuROC, area under the ROC curve; eGFR, estimated glomerular filtration rate; eGFR_Cr_, eGFR based on creatinine; eGFR_CysC_, eGFR based on cystatin C; eGFR_Cr-CysC_, eGFR based on both creatinine and cystatin C; ROC, receiver operating characteristic.

Figure 4 shows the ROC curves and AuROC values of eGFR_Cr_, eGFR_CysC_, and eGFR_Cr-CysC_ for hyperparathyroidism with hyperphosphatemia occurrence in the overall cohort and low- and high-difference groups. In the overall cohort and low-difference group, the AuROC values of eGFR_CysC_ and eGFR_Cr-CysC_ were not markedly different from that of eGFR_Cr_. However, in the high-difference group, the AuROC values of eGFR_CysC_ and eGFR_Cr-CysC_ were slightly higher than that of eGFR_Cr_ (0.677 for eGFR_CysC_ and 0.675 for eGFR_Cr-CysC_ vs. 0.658 for eGFR_Cr_).

### Comparison of AuROC values of the eGFR equations for hyperphosphatemia in the subpopulation with CKD grade 3

Table 3 shows the AuROC values of eGFR_Cr_, eGFR_CysC_, and eGFR_Cr-CysC_ in the total, low- and high-difference groups for hyperphosphatemia occurrence in patients with CKD grade 3 based on eGFR_Cr_. Similar to the results of the entire cohort (includes CKD grades 3 and 4), eGFR_CysC_ and eGFR_Cr-CysC_ showed higher AuROC values than that of eGFR_Cr_ for hyperphosphatemia in the overall and high-difference group. Moreover, the increase in AuROC values of eGFR_CysC_ and eGFR_Cr-CysC_ was more pronounced in patients with CKD grade 3 (0.719 for eGFR_Cr-CysC_ vs. 0.676 for eGFR_Cr_) compared with those of the entire cohort (0.683 for eGFR_Cr-CysC_ vs. 0.663 for eGFR_Cr_).

**Table 3:** Comparison of AuROC values of eGFR equations for hyperphosphatemia in the CKD grade 3 subpopulation.

	AuROC value (95% confidence interval)		
	eGFR_Cr_	eGFR_CysC_	eGFR_Cr-CysC_
Total (n = 391)	0.676 (0.574–0.778)	0.725 (0.639–0.810)	0.719 (0.628–0.810)
Low-difference group (n = 156)	0.852 (0.727–0.977)	0.865 (0.754–0.976)	0.862 (0.743–0.981)
High-difference group (n = 235)	0.602 (0.478–0.727)	0.651 (0.526–0.775)	0.644 (0.519–0.769)

*AuROC* Area under the receiver operating characteristic curve, *CKD* Chronic kidney disease, *eGFR* Estimated glomerular filtration rate, *eGFR*_*Cr*_ eGFR based on creatinine, *eGFR*_*CysC*_ eGFR based on cystatin C, *eGFR*_*Cr-CysC*_ eGFR based on both creatinine and cystatin C.

## Discussion

In this study, eGFR_CysC_ and eGFR_Cr-CysC_ were better indicators of hyperphosphatemia than eGFR_Cr_ was, both in the overall cohort and in the high-difference group. Similarly, eGFR_CysC_ and eGFR_Cr-CysC_ were better indicators of hyperparathyroidism with hyperphosphatemia than eGFR_Cr_ was in the high-difference group. These findings were more pronounced in patients with CKD grade 3 based on the eGFR_Cr_.

In the present study, age > 70 years and CKD grade 3 (low CKD grade) based on the eGFR_Cr_ were independent factors for a high difference in |eGFR_Cr_-eGFR_CysC_|. Male sex, history of HTN, and intact PTH levels showed statistically significant differences between the low- and high-difference groups, but these factors were not observed as significant factors in the binary logistic regression analysis. Sarcopenia increases with age, and the serum Cr level, which is affected by muscle mass, is lower in older people; therefore, the difference between eGFR_Cr_ and eGFR_CysC_ may be high in these groups^18^. Previous studies reported a positive correlation between age and eGFR difference^19–21^, consistent with the results of the present study. Whether urine PCR affects the eGFR difference is unclear; however, the degree of proteinuria tends to be low in the high-difference group^22–24^. In the present study, the urine PCR was also low in the high difference group, although the difference was not statistically significant. CysC is known to be associated with inflammation^25^; however, no significant difference was observed in C-reactive protein (CRP) levels between the low- and high-difference groups in the present study. This may be due to a low level of acute inflammation, as the participants in the present study were stable outpatients.

As CKD progresses, urinary phosphate excretion decreases, which causes hyperphosphatemia followed by secondary hyperparathyroidism through interrelated mechanisms^26,27^. In addition, the skeleton plays a very important role in the phosphorus balance. As CKD progresses, bone resorption increases and outpaces bone formation, which in turn increases the release of phosphorus into the blood. For this reason, we excluded patients taking phosphate-binding agents, calcitriol, and cinacalcet from our analysis, as these could significantly affect phosphorus and intact PTH levels. In any case, it is well-known that hyperphosphatemia and hyperparathyroidism are physiologic changes accompanying CKD progression^28^. The eGFR_CysC_ and eGFR_Cr-CysC_ were identified as more useful indicators associated with hyperphosphatemia and hyperparathyroidism with hyperphosphatemia than eGFR_Cr_ was in the overall cohort, especially in the high-difference group. This suggests that measuring and evaluating CysC in addition to Cr is necessary to accurately diagnose the patient’s current condition and predict prognosis in the clinical field in patients with risk factors, such as CKD grade 3 or old age.

Interestingly, the average serum Cr level was lower and the proportion of CKD grade 3 based on eGFR_Cr_ was higher in the high-difference group. As CKD progresses, tubular Cr secretion increases, which is known to result in underestimation of kidney function^29^. However, tubular CysC secretion rarely occurs as it is freely filtered in the glomerulus and is mostly absorbed and degraded in the proximal tubule^30^. Therefore, the risk of underestimating the GFR by CysC may be relatively lower compared with that by Cr. In patients with CKD grade 3 based on the eGFR_Cr_, the AuROC values of eGFR_CysC_ and eGFR_Cr-CysC_ improved more markedly compared with that of eGFR_Cr_. This suggests that evaluating CKD grade 3 using only eGFR_Cr_ is likely to result in a significant underestimation of kidney function.

Patients with eGFR difference > 15 ml/min/1.73 m^2^ were excluded from the present study according to a previous report which stated that a > 15 ml/min/1.73 m^2^ difference between eGFR_Cr_ and eGFR_CysC_ is probably linked to a disproportionate effect of non-GFR factors by one of the two biomarkers (Cr and CysC)^24,31^. Although many previous studies have shown that eGFR_Cr-CysC_ has the highest accuracy with mGFR, it is necessary to keep in mind that the eGFR_Cr-CysC_ equation was developed in a relatively healthy population with an average age of 47 years and an mGFR of 68 ml/min/1.73 m^2^. This study is significant in that it reveals for the first time the usefulness of eGFR_Cr-CysC_ in relation to hyperphosphatemia and hyperparathyroidism, which are physiological changes following the progression of advanced CKD.

Our study has several limitations. First, because this was a retrospective, cross-sectional study, we could not analyze the patients’ future clinical course. Second, we used inorganic phosphorus and PTH levels as indicators of physiological changes in CKD, however, the change in fibroblast growth factor 23 (FGF-23) level precedes hyperphosphatemia and is observed concurrently with PTH level elevation^32,33^. Although FGF-23 may better reflect the physiological changes in CKD, it is not yet routinely measured in clinical practice. Nevertheless, it is significant that we analyzed a large population of over 600 patients, and this is the first study to compare Cr and CysC levels in association with hyperphosphatemia and secondary hyperparathyroidism with hyperphosphatemia.

In conclusion, patients aged > 70 years and with a low CKD grade according to the eGFR_Cr_ could be at high risk for a high difference in the eGFR_Cr_ and eGFR_CysC_. Compared with that of eGFR_Cr_, the eGFR_CysC_ and eGFR_Cr-CysC_ showed a stronger association with the physiological changes of CKD progression (hyperphosphatemia and hyperparathyroidism), and the effect was particularly evident in the high-difference group and low CKD grade subpopulation. Although the usefulness of CysC is well known, in actual clinical practice many physicians still evaluate kidney function based only on Cr. Evaluating kidney function by including CysC is necessary to accurately evaluate patient kidney function and predict prognosis, at least in selected patients (such as the elderly or those with CKD grade 3) based on factors related to the high-difference of |eGFRCr-eGFRCysC|.

## Methods

### Study design

This was a single-center retrospective cross-sectional study. We used outpatient clinic data from the Clinical Data Warehouse system of Konyang University Hospital between January 2019 and February 2022. We collected the data of adult patients (≥ 19 years old) diagnosed with CKD grade 3 or 4 as defined by the Kidney Disease: Improving Global Outcomes guideline (eGFR_Cr_ categories of grade 3: 30–59 ml/min/1.73 m^2^, grade 4: 15–29 ml/min/1.73 m^2^)^34^. Serum Cr, CysC, inorganic phosphorus, calcium, and PTH levels were tested on the same day (n = 835).

**Figure 5:**
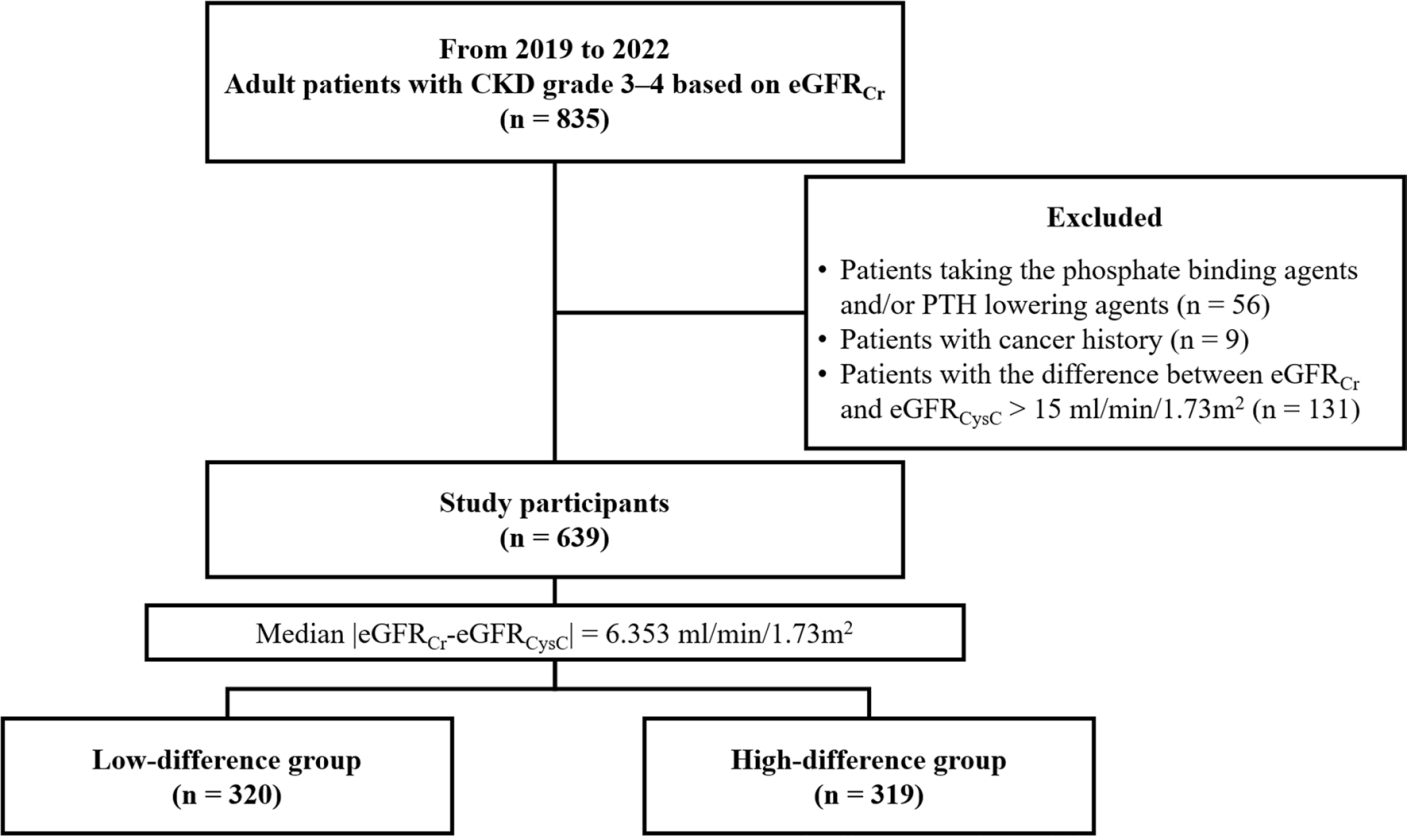
Study design. Of the 835 patients with CKD grades 3 and 4 based on the eGFR_Cr_, 196 were excluded according to the exclusion criteria, and 639 were finally included. Patients were divided into two groups based on a median |eGFR_Cr_-eGFR_CysC_| of 6.353 ml/min/1.73 m^2^. CKD, chronic kidney disease; eGFR, estimated glomerular filtration rate; eGFR_Cr_, eGFR based on creatinine; eGFR_CysC_, eGFR based on cystatin C; PTH, parathyroid hormone.

We excluded patients taking phosphate-binding agents (sevelamer, lanthanum, calcium-based phosphate binder) or PTH-lowering agents (calcitriol, cinacalcet) (n = 56), those with a history of cancer (n = 9), and those with a difference between eGFR_Cr_ and eGFR_CysC_ > 15 ml/min/1.73 m^2^ (n = 131). Therefore, 639 patients were enrolled in this study and divided into low- and high-difference groups based on the median value of |eGFR_Cr_-eGFR_CysC_|: < 6.353 ml/min/1.73 m^2^ and ≥ 6.353 ml/min/1.73 m^2^, respectively (Fig. 5).

This study was performed in accordance with the Declaration of Helsinki, and was approved by the Institutional Review Board of Konyang University Hospital (KYUH 2022-10-003). The need to obtain informed patient consent was waived by the Institutional Review Board of Konyang University Hospital (KYUH 2022-10-003) because the patient data were extracted in an anonymized form.

### Data collection

Baseline sociodemographic characteristics, including sex; age; history of HTN (systolic blood pressure ≥ 140 mmHg or diastolic blood pressure ≥ 90 mmHg or use of antihypertensive medications), diabetes mellitus (HbA1c ≥ 6.5%, fasting glucose level ≥ 126 mg/dl, or oral hypoglycemic agent or insulin administration), and cancer (patients diagnosed with the 10th Revision code of the International Classification of Diseases: C16, C18, C19, C22, C34, and C50), were obtained. We further collected information on the serum hemoglobin, albumin, CRP, uric acid levels, and urine PCR.

### ***Calculations of eGFR***_***Cr***_***, eGFR***_***CysC***_, ***and eGFR***_***Cr-CysC***_

Serum Cr and CysC levels were measured using a Beckman Coulter AU5800. Serum Cr levels were measured with the isotope dilution mass spectroscopy-traceable Jaffe method using picric acid. Serum CysC levels were measured by turbidity analysis. The CKD-EPI eGFR equations, the 2021 CKD-EPI creatinine equation (eGFR_Cr_), 2012 CKD-EPI cystatin C equation (eGFR_CysC_), and 2021 CKD-EPI creatinine-cystatin C equation (eGFR_Cr-CysC_) were as follows: eGFR_Cr_ = 142 × min (S_Cr_/κ, 1)^a^ × max (S_Cr_/κ, 1)^−1.200^ × 0.9938^age^ × (1.012 if female), eGFR_CysC_ = 133 × min (S_Cys_/0.8, 1)^−0.499^ × max (S_Cys_/0.8, 1)^−1.328^ × 0.996^age^ × (0.932 if female), and eGFR_Cr-CysC_ = 135 × min (S_Cr_/κ, 1)^b^ × max (S_Cr_/κ, 1)^−0.544^ × min (S_Cys_/0.8, 1)^−0.323^ × max(S_Cys_/0.8, 1)^−0.778^ × 0.9961^age^ × (0.963 if female). S_Cr_ is the serum Cr level; S_Cys_ is the serum CysC level; κ is 0.7 for females and 0.9 for males; a is − 0.241 for females and − 0.302 for males, and b = − 0.219 for females and − 0.144 for males^3,4,10^.

### Measurements and definition of hyperphosphatemia and hyperparathyroidism

Serum inorganic phosphorus levels were measured using a photometric ultraviolet test with molybdate using a Beckman Coulter AU5800. Serum PTH levels were measured by two-site immunoenzymatic assay using a Beckman Coulter DxI. Hyperphosphatemia was defined as a serum inorganic phosphorus level > 4.6 mg/dl, while hyperparathyroidism was defined as a serum PTH level > 65 pg/ml^35^. The AuROC values of the eGFR equations for the occurrence of hyperphosphatemia and hyperparathyroidism with hyperphosphatemia were analyzed.

### Statistical analyses

The baseline sociodemographic characteristics and laboratory findings were compared between the low- and high-difference groups. Continuous variables are expressed as the mean and standard deviation and were compared using the Student’s *t*-test. Categorical variables are expressed as numbers (percentages) and were compared using the Chi-square or Fisher’s exact test, as appropriate. Since both eGFR_Cr_ and eGFR_CysC_ showed a non-normal distribution, Spearman’s correlation analysis was performed in the overall cohort and low- and high-difference groups to analyze the correlation between eGFR_Cr_ and eGFR_CysC_. Multivariate binary logistic regression analysis was performed to delineate the relationship between the factors and the high difference in |eGFR_Cr_-eGFR_CysC_|. We compared the AuROC values between eGFR_Cr_, eGFR_CysC_, and eGFR_Cr-CysC_ to analyze the association intensity related to the occurrence of hyperphosphatemia and hyperparathyroidism with hyperphosphatemia in the overall cohort and low- and high-difference groups. A subgroup analysis was performed for patients with CKD grade 3 based on the eGFR_Cr_, and the AuROC values of the three eGFR equations were compared. Statistical significance was defined as *p* < 0.05. All statistical analyses were performed using SPSS version 23.0 (IBM Corporation, Armonk, NY, USA).

## Data Availability

The datasets generated and analyzed during the present study are available from the corresponding author on reasonable request. The data are not publicly available due to privacy or ethical restrictions.
